# Influence of 3D Printing Conditions on Physical–Mechanical Properties of Polymer Materials

**DOI:** 10.3390/polym17010043

**Published:** 2024-12-28

**Authors:** Lubomír Beníček, Martin Vašina, Pavel Hrbáček

**Affiliations:** 1 Faculty of Technology, Tomas Bata University in Zlin, Vavreckova 5669, 760 01 Zlin, Czech Republic; benicek@utb.cz (L.B.); p_hrbacek@utb.cz (P.H.); 2 Faculty of Mechanical Engineering, Department of Hydromechanics and Hydraulic Equipment, VŠB-Technical University of Ostrava, 17. listopadu 2172/15, 708 00 Ostrava-Poruba, Czech Republic

**Keywords:** 3D printing conditions, polymer materials, microscopy, three-point bend, mechanical vibration, sound absorption, light transmission

## Abstract

The popularity of 3D printing technology is rapidly increasing worldwide. It can be applied to metals, ceramics, composites, hybrids, and polymers. Three-dimensional printing has the potential to replace conventional manufacturing technologies because it is cost effective and environmentally friendly. This paper focuses on the influence of 3D printing conditions on the physical and mechanical properties of polylactic acid (PLA), poly(methyl methacrylate) (PMMA), and poly(ethylene terephthalate glycol-modified) (PETG) materials produced using Fused Deposition Modeling (FDM) technology. The impact of nozzle diameter, layer height, and printing temperature on the mechanical (i.e., bending stiffness and vibration damping) and physical (i.e., sound absorption and light transmission) properties of the studied polymer materials was investigated. It can be concluded that 3D printing conditions significantly influenced the structure and surface shape of the 3D-printed polymer samples and, consequently, their physical and mechanical properties. Therefore, it is essential to consider the type of filament used and the 3D printing conditions for specific 3D-printed material applications.

## 1. Introduction

The development of 3D printing, also known as additive manufacturing (AM), has revolutionized the manufacturing landscape since its inception in the 1980s. This transformative technology was first introduced by Charles Hull with the invention of stereolithography (SLA) in 1986 [1,2]. AM began a manufacturing revolution by offering a novel way to create physical objects directly from digital 3D models. Unlike traditional manufacturing processes, which involve removal or deformation to achieve a desired shape, AM constructs objects layer by layer. This approach enables the creation of complex geometries that are often unattainable through conventional techniques [3,4,5]. The advantages of AM include reduced material waste, accelerated prototyping, and enhanced customization capabilities [6]. AM technologies, such as fused deposition modeling (FDM), selective laser sintering (SLS), and digital light processing (DLP), are used for a wide range of materials, including polymers, metals, ceramics, composites, and hybrids [7,8,9]. The versatility of 3D printing has led to its adoption in numerous industries. In healthcare, it produces custom prostheses, implants, biodegradable polymers, and composites for bone tissue engineering [10,11]. The automotive and aerospace industries benefit from the ability to prototype and produce complex, lightweight components rapidly. Additionally, AM plays a significant role in the food industry (e.g., 3D printing of chocolate, pasta noodles, baked goods, and meat alternatives) [12,13,14,15], consumer goods (e.g., jewelry, home furniture, decoration, sports equipment, toys, and games) [16,17], soft robotic actuators [18], education [19], electronics, fashion, architecture, and construction [2,20]. Over the past few decades, 3D printing has evolved from a tool primarily used for rapid prototyping to a method of creating functional end-use products. Its capabilities for mass customization, shorter production times, and minimal environmental impact have made it an attractive alternative to conventional manufacturing technologies. The potential of 3D printing to revolutionize industries and supply chains remains immense, promising greater efficiency, sustainability, and innovation [21]. However, it is crucial to consider the specific parameters of 3D printing, as they significantly affect the mechanical properties, dimensional accuracy, and surface quality of the printed samples [22]. Different factors, such as filament and nozzle diameter, printing speed and temperature, layer thickness, print orientation, and post-processing techniques, influence the final properties of 3D-printed samples.

The influence of various 3D printing parameters, such as nozzle diameter, infill orientation, layer height, and printing temperature, on the tensile and flexural properties of glass fiber-reinforced nylon 6/66 composites was examined in [23]. The study found that the highest mechanical stiffness was achieved with a 0° infill orientation, using a high layer height and printing temperature. At the same time, the nozzle diameter did not show a consistent impact on the mechanical properties. Shergill et al. [24] investigated the effect of layer thickness on the mechanical properties of 3D-printed specimens made from PLA, ABS, and PETG. They found that increasing layer thickness decreased ultimate tensile strength and breaking strain for all materials. However, PLA specimens exhibited a more pronounced reduction in tensile strength and breaking strain due to hydrolysis. At the same time, PETG specimens exhibited a smaller decline in mechanical properties with increased layer thickness. Also, layer thickness did not significantly affect Young’s modulus for any materials. It was observed in [25] that decreasing layer thickness increased the proportionality limit and elastic modulus of investigated PLA-Cg+ specimens. When the loading axis was angled relative to the filament deposition direction, the specimens exhibited lower proportionality limits and mechanical stiffness. Another study [22] found that increasing layer thickness could reduce the tensile strength of ABS and PETG samples by up to 20%. In addition, a higher printing angle reduced tensile strength by approximately 12%.

The impact of build orientation, infill pattern, and infill density on the surface strain of 3D-printed PLA specimens was examined in [26]. The study found that specimens printed in an on-edge orientation (0°) exhibited significantly lower tensile strength and Young’s modulus than those in an upright orientation (90°), highlighting anisotropic behavior. Further research in [27] showed that on-edge-oriented PLA specimens exhibited optimal mechanical properties, including tensile strength, flexural strength, stiffness, and ductility, compared to flat and upright orientations. Overall, better mechanical performance was achieved with lower layer thickness and higher feed rates. However, ductility decreased as both parameters increased. Hasan et al. [28] confirmed that the tensile strength of 3D-printed PLA specimens fabricated by FDM increased when the rasters were oriented parallel to the applied load and with higher fill density and lower layer thickness. However, no significant influence of raster orientation on the compression properties of cylindrical samples printed from PA12 powder using a desktop SLS 3D printer was observed [29]. Additionally, the highest geometric and dimensional accuracy was noted in samples produced with the largest diameter of 9 mm. The impact of vertical and horizontal print orientations on the bending fatigue properties of ABS and PLA polymer samples was investigated in [30]. The study found that vertical specimens exhibited a shorter fatigue lifetime than horizontal samples for both materials, particularly under lower stress levels. Moreover, PLA samples demonstrated a longer fatigue lifetime than ABS under the same loading conditions. Shaik et al. [31] investigated the mechanical properties of PLA specimens printed in the longitudinal and transverse directions using a customized autoclave. The printing process was conducted under atmospheres of compressed air (0 to 20 bar) and nitrogen (5 bar) at an autoclave temperature of 50 °C. They found that preheating the autoclave before printing and applying pressure during the process significantly improved layer consolidation. This was achieved by increasing surface contact between layers, which enhanced the yield strength, Young’s modulus, and impact strength of the PLA samples.

The influence of printing temperature and annealing conditions on the tensile properties of 3D-printed PLA samples was studied in [32]. The study concluded that increasing the printing temperature enhanced tensile strength and Young’s modulus. However, it had a negligible effect on elongation at break. Additionally, prolonged annealing time was found to reduce the tensile properties of the PLA samples, likely due to stress build-up. The impact of heat treatment on the mechanical strength of PLA samples was investigated in [33]. The samples were heat-treated at temperatures ranging from 30 °C to 130 °C in 10 °C increments for 1 h, then cooled to room temperature in an oven. The heat-treated samples exhibited higher Young’s modulus and mechanical stiffness, resulting in significantly less deformation compared to untreated samples. In addition, the heat treatment changed the geometry of the samples, leading to a reduction in length and weight. Glowacki et al. [34] examined the effects of thermal shocks on the low-cycle fatigue behavior of 3D-printed ABS, acrylonitrile styrene acrylate (ASA), high-impact polystyrene (HIPS), and PLA samples that underwent temperature cycling from ambient temperature to −20 °C, followed by heating to 70 °C. The results showed reduced fatigue life for ASA and HIPS materials, while PLA exhibited increased durability. In addition, ABS resisted the environmental effects of temperature shocks. This property makes it the most suitable material for parts exposed to humidity and temperature fluctuations.

Three-dimensional printing conditions significantly affect the surface quality of 3D-printed samples. As reported in [35], the surface roughness of PLA samples generally increases with layer thickness, whereas temperature and printing speed have minimal impact on roughness. For ABS samples, ultrasonic strengthening proved more effective in reducing surface roughness. Additionally, ultrasonic strengthening significantly increases their tensile strength and Young’s modulus [36]. The study [37] further demonstrated that ultrasonic vibration improves the tensile, bending, and dynamic mechanical properties of ABS and PLA polymers.

The properties of 3D-printed materials can also be modified through reinforcement. Ning et al. [38] found that carbon fiber reinforcement significantly influenced the mechanical properties of 3D-printed ABS specimens. The carbon fiber-reinforced plastic (CFRP) specimen with 150 μm fibers demonstrated higher tensile strength and Young’s modulus than specimens with 100 μm fibers. However, the specimen reinforced with 150 μm fibers exhibited lower toughness and ductility than the one reinforced with 100 μm fibers. The CFRP composite with five wt% carbon fiber content also showed greater flexural and tensile stiffness than pure ABS. Similarly, carbon fiber-reinforced 3D-printed PLA specimens exhibited significantly higher tensile, bending, and compression stiffness than pure PLA samples [39].

The mechanical properties of PLA-based composite materials filled with bronze powder, copper powder, wood flour, and carbon nanotubes were compared to those of hot-pressed samples [40]. Tensile tests revealed that the elongation at break and yield strength of the 3D-printed samples were reduced by 15–60% compared to hot-pressed samples. Young’s modulus was also higher for PLA-based composites than for pure PLA in both 3D-printed and hot-pressed forms. Ahn et al. [41] compared the mechanical properties of ABS samples manufactured using FDM and injection molding technologies. They found that 3D-printed ABS specimens exhibited 10–73% lower tensile strength and 80–90% lower compressive strength than injection-molded specimens.

As already mentioned, many researchers have investigated the influence of 3D printing conditions on various properties of materials produced by this technology. This study investigates the influence of 3D printing conditions on the physical and mechanical properties of PLA, PMMA, and PETG polymer materials. Specifically, this paper examines the effects of nozzle diameter, layer height, and printing temperature on the bending, vibration damping, sound absorption, and light transmission properties of 3D-printed polymer materials produced using the FDM technique. In addition, the individual layers of the manufactured 3D-printed samples were alternately applied to create a cross-layer pattern. To the authors’ knowledge, no relevant studies have been published on these specific properties of 3D-printed polymer materials. The findings presented in this paper could help optimize 3D printing parameters to enhance performance and reduce operating costs in practical applications.

## 2. Materials and Methods

### 2.1. Production of 3D-Printed Polymer Samples

Polylactic acid (PLA), poly(methyl methacrylate) (PMMA), and poly(ethylene terephthalate glycol-modified) (PETG) filaments were used as primary materials to produce 3D-printed samples to investigate their physical–mechanical properties. The basic parameters of these filaments, such as diameter (*d*), Young’s modulus of elasticity (*E*), density (*ρ*), printing temperature (*T*_1_), and bed temperature (*T*_2_), are listed in Table 1.

**Figure 1:**
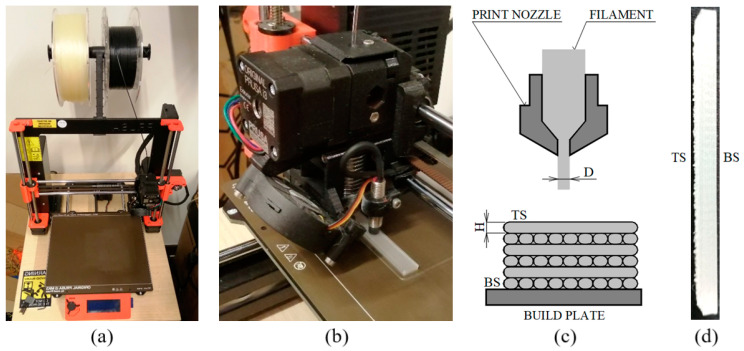
Production of 3D-printed samples: (**a**) view of Original Prusa i3 MK3 3D printer; (**b**) view of the production of a 3D-printed sample using the Original Prusa i3 MK3 3D printer; (**c**) schematic of the alternating layer deposition during the 3D printing process (TS—sample’s top side, BS—sample’s bottom side); (**d**) example of the produced 3D-printed polymer sample.

The 3D printing process was performed on an Original Prusa i3 MK3 (Prusa Research Inc., Prague, Czech Republic) 3D printer (see Figure 1a) using the FDM technique. A detailed view of the 3D-printed sample production is shown in Figure 1b. The individual layers of the 3D-printed samples were deposited alternately, with each layer rotated by 90 degrees relative to the previous one, resulting in a cross-layer pattern, as depicted in Figure 1c. A photograph of the 3D-printed sample produced in this manner is shown in Figure 1d. It can be observed that the top side (TS) of the sample exhibited significantly greater surface irregularities compared to its bottom side (BS).

Careful selection of 3D printing parameters is essential to achieve optimum print quality, mechanical properties, and overall performance of final products. Various factors affect the properties of 3D-printed objects, and understanding these parameters can significantly enhance the efficiency of the additive manufacturing process. Key parameters consider layer height, nozzle diameter, printing temperature, printing time, infill density, and print orientation.

The designation and 3D printing parameters of the manufactured 3D-printed samples are given in Table 2. The 3D-printed samples were produced using two different nozzle diameters, *D* (0.8 mm and 0.4 mm), two-layer heights, *H* (0.4 mm and 0.2 mm), and at two printing temperatures, *T*_1_. A nozzle diameter of 0.4 mm, the most commonly used size among 3D printing users due to its middle solution between precision and printing time [42,43], was used to produce the 3D-printed samples. Additionally, a second nozzle diameter of 0.8 mm was chosen for faster 3D printing. Furthermore, the 3D printing FDM technique typically uses layer heights between 0.1 mm and 0.4 mm [44,45]. A layer height of 0.2 mm was chosen as the recommended standard for FDM 3D printing due to its optimal ratio between quality and printing efficiency [46,47]. A second layer height of 0.4 mm was chosen to accelerate the printing process further. The first printing temperature was selected based on the manufacturer’s recommendation, as detailed in Table 1. The second selected printing temperature was 20 °C higher than the first recommended for each polymer type. This study also aimed to verify the influence of temperature deviation from its recommended value on 3D printing properties by increasing the temperature, focusing on the impact of lower viscosity and higher fluidity. Table 2 also shows the density (*ρ_s_*) values of the tested 3D-printed samples determined according to ISO 1183-1 method A [48].

These parameters were chosen to systematically investigate the influence of different 3D printing settings on the physical and mechanical properties of the studied polymeric materials. The aim was to identify trends and establish relationships between print quality, material properties, and production efficiency.

### 2.2. Measurement Methodology

#### 2.2.1. Microscopy

The surfaces of the 3D-printed specimens and microtome cuts were analyzed using a Keyence VHX-7100 digital microscope (Keyence Corporation, Osaka, Japan). The microscopic analysis was conducted at 80× magnification, which allowed a detailed view of the specimens’ surface properties. Microtome cuts were prepared using a Leica RM2255 rotary microtome with a thickness of 40 microns (Leica Microsystems, Wetzlar, Germany).

#### 2.2.2. Three-Point Bending Testing

**Figure 2:**
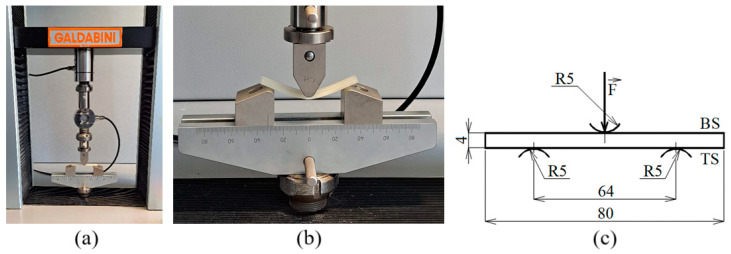
Three-point bending tests: (**a**) view of the Galdabini Quasar 25 testing machine; (**b**) detailed view of the bending test process; (**c**) illustration of the three-point bending method (TS—sample’s top side, BS—sample’s bottom side, dimensions in mm).

Static mechanical properties, including the bending modulus of elasticity (*E_B_*), maximum bending stress (*σ_max_*), strain at maximum force (*ε_Fmax_*) and at break (*ε_break_*), and absorbed energy at maximum bending stress (*E_σmax_*) and at break (*E_break_*), were evaluated using destructive three-point bending tests according to the EN ISO 178:2019 (method A) standard [49]. These tests were conducted using a Galdabini Quasar 25 universal testing machine (Galdabini Cesare S.p.A., Cardano al Campo, Italy), which has a force capacity of 25 kN and a load cell rated at 1 kN. Figure 2 presents a visual representation of the Galdabini Quasar 25 testing machine, a detailed view of the bending stress of a specific 3D-printed sample, and the three-point bending testing method. The tested samples were positioned centrally on supports spaced 64 mm apart. A 5 mm rounded push rod applied a force *F* perpendicular to the sample’s bottom side (BS) obtained through the 3D printing process, as shown in Figure 2c. A 20 mm/min test speed was maintained until a deflection of 20 mm was reached. The dimensions of the tested block articles were 80 mm × 10 mm × 4 mm (length × width × thickness). Each measurement was repeated three times at an ambient temperature of 21 °C, and the average values of the above quantities and their standard deviations were subsequently calculated.

#### 2.2.3. Vibration Damping Testing

The mechanical vibration damping properties of the tested 3D-printed polymer samples were compared using the displacement transmissibility *T_d_* (−), which is defined for the basic linear single-degree-of-freedom (SDOF) system by the equation [50,51]:

(1) $$T_{d}=\frac{X}{Y}=\sqrt{\frac{k^{2}+{\left(c\omega \right)}^{2}}{{\left(k-m\omega^{2}\right)}^{2}+{\left(c\omega \right)}^{2}}}=\sqrt{\frac{1+{\left(2\zeta r\right)}^{2}}{{\left(1-r^{2}\right)}^{2}+{\left(2\zeta r\right)}^{2}}}$$

where *X* (m) is the displacement amplitude on the output side of the tested sample, *Y* (m) is the displacement amplitude on the input side of the tested sample, *k* (N·m^−1^) is the material stiffness, *c* (N·s m^−1^) is the viscous damping coefficient, *ω* (rad·s^−1^) is the frequency of oscillation, *m* (kg) is the mass, *ζ* (−) is the damping ratio, and *r* (−) is the frequency ratio.

There are three different types of mechanical vibrations based on displacement transmissibility: damped (*T_d_* < 1), undamped (*T_d_* = 1), and resonance (*T_d_* > 1) mechanical vibrations. Using the condition *dT_d_*/*dζ* = 0 in Equation (1), is it possible to obtain the frequency ratio *r*_0_ at which the displacement transmissibility reaches a local extremum (i.e., its maximum value *T_dmax_*), specifically at the first resonance frequency *f_R_*_1_ [52,53]:

(2) $$r_{0}=\frac{\sqrt{\sqrt{1+8\zeta^{2}}-1}}{2\zeta }$$

It is evident from Equation (2) that the frequency ratio *r*_0_ generally decreases with increasing damping ratio *ζ* (or decreasing mechanical stiffness *k*) [53].

Experimental measurements of the displacement transmissibility of the investigated 3D-printed polymer specimens were conducted using the method of harmonically excited mechanical vibrations within the frequency range of 2–1600 Hz. The measuring apparatus (Brüel & Kjær, Nærum, Denmark) consisted of a mini-shaker (BK 4810), a signal PULSE multi-analyzer (BK 3560-B-030), and a power amplifier (BK 2706). For harmonically excited mechanical vibrations, Equation (2) can also be modified as follows:

(3) $$T_{d}=\frac{A_{X}}{A_{Y}}$$

where *A* (m·s^−2^) is the acceleration amplitude on either the output (*X*) and input (*Y*) sides of the tested specimen. The displacement transmissibility was determined from Equation (3) based on the measured acceleration amplitudes recorded using BK 4393 piezoelectric accelerometers (Brüel & Kjær, Nærum, Denmark). The tested block articles had dimensions of 60 mm × 60 mm × 2 mm (length × width × thickness) and were loaded with an inertial mass of 90 g, which was placed on the top side (TS) of the tested harmonically loaded 3D-printed samples. Each measurement was repeated five times under ambient conditions at a temperature of 22 °C.

#### 2.2.4. Sound Absorption Properties

Sound absorption properties of materials are expressed by the sound absorption coefficient *α* (−), which is defined as follows [54]:

(4) $$\alpha =1-\frac{E_{R}}{E_{I}}=\frac{E_{A}}{E_{I}}$$

where *E_R_* (J) is the reflected sound energy, *E_I_* (J) is the incident sound energy, and *E_A_* (J) is the absorbed sound energy. A material’s ability to absorb sound is affected by various factors, including the frequency of incident acoustic waves, material structure, thickness, density, surface shape, and temperature [55].

Frequency dependencies of the normal incidence sound absorption coefficient *α* of the tested 3D-printed polymer specimens were determined experimentally using a two-microphone acoustic impedance tube (BK 4206) in conjunction with a signal PULSE multi-analyzer (BK 3560-B-030) and a power amplifier (BK 2706) in the frequency range from 250 to 4000 Hz (Brüel & Kjær, Nærum, Denmark). In this case, acoustic waves were propagated perpendicular to the top side (TS) of the 3D-printed samples. All samples were cylindrical, with an outer diameter of 29 mm and a thickness of 10 mm. All measurements were conducted at an ambient temperature of 23 °C.

Based on the partial standing wave principle, frequency dependencies of the sound absorption coefficient of the investigated polymer specimens were determined using the two-microphone transfer function method according to ISO 10534-2 [56] standard. The normal incidence sound absorption coefficient *α* is defined by the following equation [57,58]:

(5) $$\alpha =1-{\left|R\right|}^{2}=1-{\left|\frac{Z_{s}-\rho_{0}\cdot c_{0}}{Z_{s}+\rho_{0}\cdot c_{0}}\right|}^{2}$$

where *R* (−) is the normal incidence reflection factor, *Z_s_* (kg·m^2^·s^−1^) is the surface acoustic impedance, *ρ*_0_ (kg·m^−3^) is the air density, and *c*_0_ (kg·m^−3^) is the sound speed in the air.

#### 2.2.5. Light Transmission Properties

The ability to transmit light of a light-transparent material depends not only on its type but also on various other factors, such as its color, structure, surface shape and contamination, thickness, density, refractive index, temperature, light wavelength, and angle of light incidence [59,60,61,62]. The light transmission properties of the studied 3D-printed polymer specimens were investigated based on their light transmittance and transmission haze.

The ability of the investigated polymer samples to transmit diffuse daylight is characterized by the diffuse light transmittance *T* (−), which is given by the following equation [63]:

(6) $$T=\frac{\Phi_{T}}{\Phi_{I}}$$

where *Φ_T_* (W) is the transmitted luminous flux, and *Φ_I_* (W) is the incident luminous flux. The diffuse light transmittance *T* was experimentally determined based on the ČSN 360011-2 standard [64] by the illumination ratio method, according to the following equation:

(7) $$T=\frac{E_{T}}{E_{I}}$$

where *E_T_* (lx) is the illuminance measured behind the embedded polymer sample, and *E_I_* (lx) is the incident illuminance measured without the embedded polymer sample (i.e., after its removal). The experimental measurements of the light transmission properties of the investigated polymer samples were conducted using a Voltcraft MS-1300 luxmeter (Voltcraft, Hirschau, Germany). These measurements were performed during the propagation of diffused daylight through the samples, specifically from the top side (TS) to the bottom side (BS). They were conducted in the shade under clear skies during the summer around midday to ensure the highest possible accuracy in determining the diffuse light transmittance. The dimensions of the unpolluted test specimens were 60 mm × 60 mm × 2 mm (length × width × thickness). Each measurement was repeated 20 times at an ambient temperature of (24 ± 2) °C. The mean values and standard deviations of the light transmittance were then determined.

The haze *H* (%) is the percent of transmitted light that is scattered so that its direction deviates more than 2.5° and is defined by the following equation [65,66]:

(8) $$H=\frac{{\left(I_{t}\right)}_{2.5}^{90}}{I_{t}}\cdot 100$$

where *I_t_* (W/m^2^) is the intensity of the transmitted light, and ${\left(I_{t}\right)}_{2.5}^{90}$ (W/m^2^) is the intensity of a part of the transmitted light with a scattering angle greater than 2.5° as it passes through the tested material sample. The haze *H* was experimentally determined according to ASTM D1003 Procedure B (Spectrophotometer) standard [67] using an UltraScan Pro D65 spectrophotometer (HunterLab, Reston, VA, USA). Similarly, as in the case of the light transmission, the unpolluted tested samples measuring 60 mm × 60 mm × 2 mm (length × width × thickness) were used for the haze measurements. Each measurement was repeated 5 times at a temperature of (23 ± 1) °C. The mean values and standard deviations of the haze were subsequently determined.

## 3. Results and Discussion

### 3.1. Microscopy Analysis of 3D-Printed Polymer Samples

**Figure 3:**
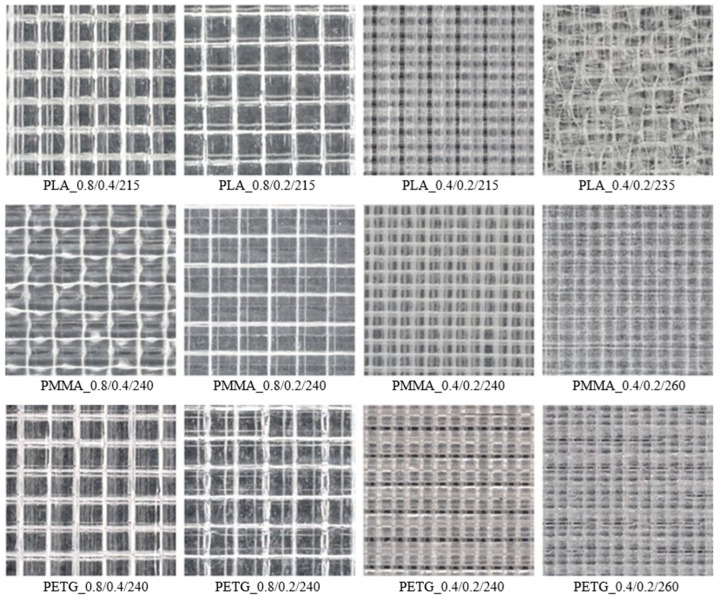
Microscopic images of surface shapes of the investigated 3D-printed polymer specimens with 5 mm × 5 mm plan dimensions.

Figure 3 depicts microscopic images of the surface shapes of the studied 3D-printed polymer specimens, with plan dimensions of 5 mm × 5 mm, manufactured with different nozzle diameters, layer heights, and printing temperatures. These images demonstrate the principle of alternating layers during the 3D printing process, with each layer rotated 90 degrees relative to the previous one, as shown graphically in Figure 1c. These images also illustrate that the 3D printing conditions significantly influenced the surface shape and structure of the investigated polymer samples, which affected their physical and mechanical properties.

**Figure 4:**
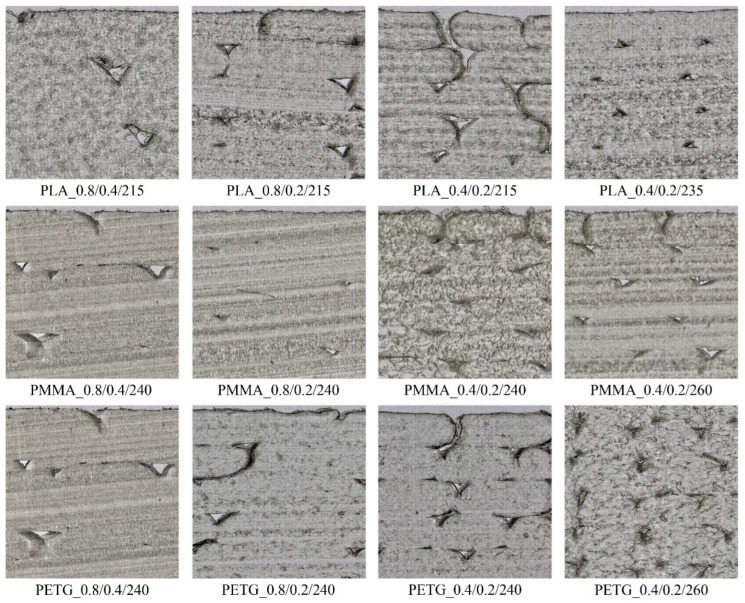
Microscopic images of structural sections of the investigated 3D-printed polymer specimens with plan dimensions of 1 mm × 1 mm.

Microscopic images of structural sections of the investigated 3D-printed polymer samples are shown in Figure 4. The microtome sections revealed significant insights into the layer adhesion quality of the samples. Improved layer adhesion was observed with increased printing temperature and thicker nozzle diameters. Samples with smaller layer height exhibited a higher presence of internal voids. Thinner layers may not bond as effectively, leading to an increase in voids. Although samples with larger nozzle diameters and thicker layers had larger internal voids, their overall quantity was lower than those with thinner layers and smaller nozzle diameters. This suggests that larger nozzle diameters and thicker layers enhance bonding, thereby reducing the number of voids. Additionally, increasing the printing temperature improved layer adhesion and reduced the number of internal voids for the same nozzle diameter and layer height. This highlights the importance of optimizing the printing temperature to enhance the quality of the printed material. Both larger nozzle diameters and thicker printing layers contributed to better layer adhesion, indicating that adjusting these parameters can significantly improve the structural integrity of printed samples. These observations are also consistent with the density values (*ρ_s_*) of the samples presented in Table 2.

### 3.2. Bending Tests

**Figure 5:**
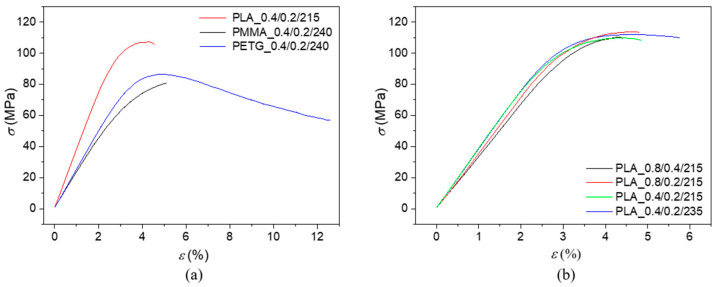
Stress–strain dependencies under uniaxial bending tests of the investigated polymer specimens: (**a**) effect of the investigated polymer material, nozzle diameter *D* = 0.4 mm, layer height *H* = 0.2 mm; (**b**) effect of 3D printing conditions on the investigated PLA specimens.

**Figure 6:**
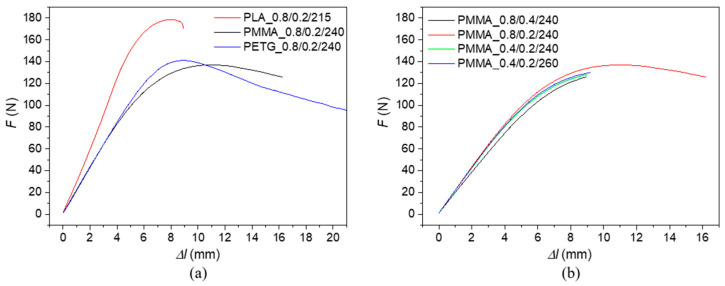
Load-deflection dependencies under uniaxial bending tests of the investigated polymer specimens: (**a**) effect of the investigated polymer material, nozzle diameter *D* = 0.8 mm, layer height *H* = 0.2 mm; (**b**) effect of 3D printing conditions on the investigated PMMA specimens.

Examples of the experimentally measured uniaxial bending responses of the investigated 3D-printed polymer samples are displayed in Figure 5 and Figure 6. Figure 5a shows the stress–strain dependencies of three 3D-printed polymer specimens, all manufactured with a nozzle diameter of 0.4 mm and a layer height of 0.2 mm. Similarly, the stress–strain dependencies of the PLA specimens produced under different 3D printing conditions are depicted in Figure 5b. The load-deflection (*Δl*) characteristics of three different 3D-printed polymer specimens manufactured with a nozzle diameter of 0.8 mm and a layer height of 0.2 mm, as well as the effect of 3D printing conditions on the load-deflection dependencies of the PLA specimens, are shown in Figure 6a,b.

Experimentally measured uniaxial bending responses were used to evaluate the bending modulus of elasticity (*E_B_*), maximum bending stress (*σ_max_*), strain at maximum force (*ε_Fmax_*) and at break (*ε_break_*), and absorbed energy at maximum bending stress (*E_σmax_*) and at break (*E_break_*) that are proportional to the area under the load-deflection curves (see Figure 6). The mean values and corresponding standard deviations of the quantities mentioned above are given in Table 3.

The bending tests showed that the PLA samples exhibited the highest mechanical stiffness, as indicated by the highest measured bending modulus values and maximum bending stress, regardless of the 3D printing conditions. These findings are consistent with Young’s modulus values of the filaments used to produce the 3D-printed samples, as shown in Table 1. The other types of 3D-printed materials, i.e., the investigated PMMA and PETG samples, exhibited significantly lower bending modulus (30 to 39%) and, consequently, lower bending stiffness than the PLA samples due to their different macromolecular structure. The bending modulus of elasticity increased, regardless of the sample type, with a decrease in nozzle diameter and layer height. It confirms that small nozzle diameters and layer heights ensure more efficient melting and interlayer adhesion. It minimizes the melt spread by decreasing printed layer thickness, even at lower printing temperatures [68]. The impact of the printing temperature on the mechanical stiffness of the investigated 3D-printed samples requires further clarification. For the tested PLA and PMMA samples, the bending modulus increased with increasing printing temperature. The opposite phenomenon, i.e., a decrease in bending modulus of elasticity (from 2441 to 2385 MPa) with increasing printing temperature, was observed for the studied PETG samples. Higher temperatures reduce the viscosity of the material, causing the polymer to melt more easily. As a result, the height of the printed layer is not maintained (see Figure 3), which affects the mechanical properties of the materials [68].

It is also evident (see Table 3) that the effect of sample type and printing conditions on the strain at maximum force (*ε_Fmax_*) was insignificant. However, significant changes in behavior were observed during bending tests for the strain at break (*ε_break_*). The maximum strain at break (9.2%) was recorded for the PMMA sample printed with a nozzle diameter of 0.8 mm and a layer height of 0.2 mm. As shown in Figure 4, the higher bending resistance at break of the PMMA_0.8/0.2/240 sample is attributed to its structure compared to the PMMA_0.8/0.4/240 sample, specifically better layer cohesion, leading to improved bonding between individual layers, better stress distribution, more flexible behavior, and lower material porosity. Therefore, the PMMA_0.8/0.2/240 sample absorbed more energy at both maximum bending stress and at the point of break.

In addition, the measurement results (see Table 3) showed that none of the tested PETG samples reached fracture at a maximum deflection of 20 mm during the bending test. In such tests, ductility refers to a material’s ability to undergo plastic deformation under bending stress before failure. This property is crucial in engineering and manufacturing, as it determines a material’s suitability for specific applications and ability to absorb mechanical overloads [69].

It can be stated that the 3D cross-layer deposition printing technique used for specimen production (see Figure 1c) enhanced the bending properties (i.e., bending modulus of elasticity and maximum bending stress) of the tested polymer (PLA, PMMA, and PETG) materials compared to conventionally 3D-printed samples produced with different raster angle orientations [70,71,72,73,74].

### 3.3. Vibration Damping Properties

**Figure 7:**
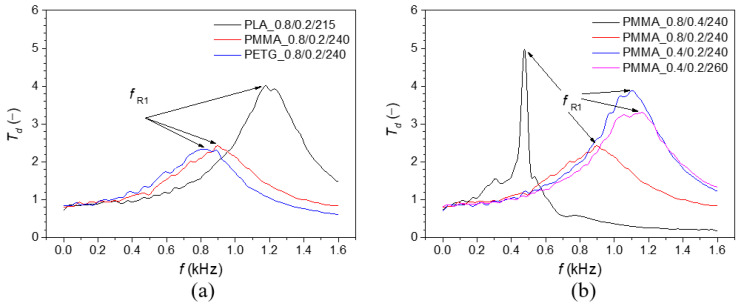
Frequency dependencies of the displacement transmissibility: (**a**) 3D-printed polymer samples with a nozzle diameter of *D* = 0.8 mm, a layer height of *H* = 0.2 mm, and loaded with an inertial mass of *m_i_* = 90 g; (**b**) effect of 3D printing conditions of the investigated PMMA samples loaded with an inertial mass of *m_i_* = 90 g.

The frequency dependencies of the displacement transmissibility *T_d_* of the investigated 3D-printed samples loaded with an inertial mass of 90 g are depicted in Figure 7. Figure 7a compares the mechanical vibration damping properties of the studied polymer materials under the same printing conditions, specifically for samples printed at a nozzle diameter of 0.8 mm and a layer height of 0.2 mm with lower printing temperatures. The lowest material’s ability to damp mechanical vibrations was found for the tested PLA specimen, characterized by resonance mechanical vibrations (i.e., *T_d_* > 1) over the measured frequency range. The ability to dampen mechanical vibrations (i.e., *T_d_* < 1) was generally observed at higher excitation frequencies for the PMMA (i.e., at *f* > 1418 Hz) and PETG (i.e., at *f* > 1220 Hz) specimens. This phenomenon is reflected in the first resonance frequency (*f_R_*_1_) peak position, which generally decreases with the decreasing sample stiffness *k* (or the increasing damping ratio *ζ*), as given in Equation (2). The mean values and corresponding standard deviations of the first resonance frequency for the tested polymer samples are shown in Table 4.

Based on the first resonance frequencies that were experimentally obtained, it was observed that the investigated PLA specimens generally exhibited higher dynamic mechanical stiffness compared to the PMMA and PETG specimens. In contrast, the PLA sample exhibited the lowest first resonance frequency (749 Hz) and, consequently, the lowest dynamic mechanical stiffness at the increased printing temperature. In comparison, the tested PMMA and PETG specimens exhibited first resonance frequencies of 1125 Hz and 1299 Hz, respectively. It is related to the significant structural degradation of the PLA_0.4/0.2/235 sample at the increased printing temperature (see its microscopic structure in Figure 3). Consequently, the higher printing temperature of the PLA_0.4/0.2/235 sample resulted in a significant decrease in the first resonance frequency, improving its ability to dampen mechanical vibrations. The higher printing temperature also decreased the first resonance frequency (i.e., from 1351 to 1299 Hz) and thus enhanced the vibration damping properties of the studied PETG sample, as shown in Table 4. The opposite effect of printing temperature on the first resonance frequency was observed for the tested PMMA specimen, as shown in Figure 7b. The vibration damping properties of the PMMA samples generally decreased with increasing printing temperature and decreasing nozzle diameter and layer height, as reflected in the increase in the first resonance frequency from 480 Hz to 1125 Hz (see Table 4).

It can be concluded from the displacement transmissibility measurements that the studied 3D-printed PLA specimens exhibited higher first resonance frequencies and, consequently, higher mechanical stiffness compared to the other materials (i.e., PMMA and PETG). It corresponds with the superior mechanical properties of the PLA material, characterized by higher values of Young’s modulus and bending modulus of elasticity, as shown in Table 1 and Table 3. Only in the case of the increased printing temperature (i.e., 235 °C) during the 3D printing process did the mechanically degraded structure of the PLA sample lead to higher internal friction under dynamic loading and, consequently, higher conversion of the input mechanical energy into heat [75]. This was accompanied by a shift in the first resonance frequency peak position to a lower value, resulting in improved vibration damping properties of the PLA_0.4/0.2/235 sample compared to those printed at a lower temperature (i.e., 215 °C). As a result, this PLA sample’s dynamic mechanical stiffness was lower than the other samples (i.e., PMMA_0.4/0.2/260 and PETG_0.4/0.2/260) produced at higher printing temperatures.

### 3.4. Sound Absorption Properties

As mentioned above, the sound absorption properties of materials depend on many parameters. The applied conditions of the 3D printing process also influence their ability to absorb sound.

**Figure 8:**
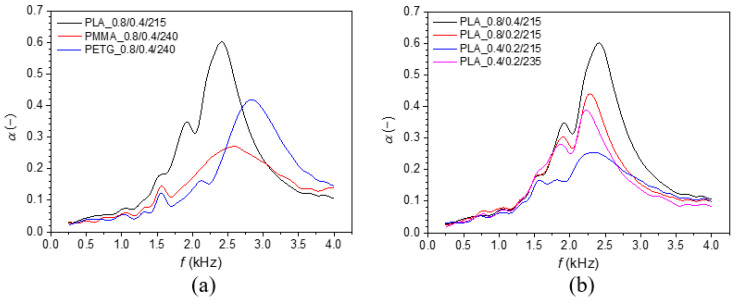
Frequency dependencies of the sound absorption coefficient: (**a**) 3D-printed polymer samples with a nozzle diameter *D* = 0.8 mm and a layer height *H* = 0.4 mm; (**b**) effect of 3D printing conditions of the investigated PLA samples.

The frequency dependencies of the investigated material specimens, printed with a nozzle diameter of 0.8 mm and a layer height of 0.4 mm, are compared in Figure 8a. The best sound absorption properties were generally observed for the 3D-printed PLA specimen, particularly in the frequency range from 1000 to 2700 Hz. This is also reflected in the highest values of the mean sound absorption coefficient (*α_m_*) and the noise reduction coefficient (*NRC*), regardless of the 3D printing conditions, as shown in Table 5. This improvement was likely due to the greater surface irregularities (see Figure 3) created during 3D printing of PLA samples compared to other studied materials. These irregularities result in multiple reflections of incident acoustic waves, leading to a higher conversion of acoustic energy into heat. Figure 8a also shows that the investigated 3D-printed PETG sample exhibited better sound absorption at higher excitation frequencies (i.e., *f* > 2700 Hz) than the other tested 3D-printed samples. Additionally, the sound absorption properties of all tested 3D-printed samples are generally low and very similar at lower excitation frequencies. The experimentally determined values of the mean sound absorption coefficient (*α_m_*), noise reduction coefficient (*NRC*), maximum sound absorption coefficient (*α_max_*), and the corresponding excitation frequency (*f_max_*) are summarized in Table 5.

The influence of 3D printing conditions on the sound absorption performance of the investigated PLA samples is shown in Figure 8b. The sound absorption properties of these samples generally decreased with the decrease in the nozzle diameter and the layer height during the 3D printing process at the printing temperature of 215 °C. However, the increased printing temperature (i.e., 235 °C) at the lowest nozzle diameter (i.e., 0.4 mm) and layer height (i.e., 0.2 mm) led subsequently to improved sound absorption of the studied PLA sample. Similar to the vibration damping measurements, this phenomenon is again caused by structural degradation at the increased printing temperature (see the microscopic structure of the PLA_0.4/0.2/235 sample in Figure 3). This results in a highly irregular surface structure that leads to multiple reflections of acoustic waves on contact with the sample surface, thereby increasing sound absorption. It is also evident that the ability to absorb sound of the investigated PMMA and PETG samples decreased not only with reducing nozzle diameter and layer height but also with increasing printing temperature. As a result, their surfaces became gradually smoother with decreasing nozzle diameter, layer height, and printing temperature, as depicted in Figure 3. This phenomenon was also confirmed by the decreasing value of the mean sound absorption coefficient (*α_m_*), as shown in Table 5.

The applied 3D printing conditions significantly influenced the surface quality (see Figure 3) of the manufactured 3D-printed samples and, thus, their sound absorption properties. Greater surface roughness (or unevenness) improved acoustic resistance, leading to an increase in the sound absorption coefficient [76]. Therefore, the ability to absorb the sound of the investigated 3D-printed samples decreased with decreasing nozzle diameter and layer height and with increasing printing temperature for the studied PMMA and PETG samples. In contrast, higher printing temperatures improved the sound absorption properties of the investigated PLA sample.

### 3.5. Light Transmission Properties

As mentioned above, the light transmission properties of the tested 3D-printed material samples were evaluated based on their light transmittance and haze during the propagation of diffused light through the samples.

#### 3.5.1. Light Transmittance

The experimentally measured mean values of the light transmittance and their standard deviations are presented in Table 6. The material’s ability to transmit diffuse daylight depends on the filament type used for producing the 3D-printed samples and the applied 3D printing conditions. It can be stated that the light transmission properties of the tested 3D-printed material samples generally increased with larger nozzle diameters and layer heights for all material types, as shown in Table 6. This was caused mainly by the surface shape and structure of the samples, which are significantly influenced by the applied 3D printing conditions. In general, larger nozzle diameter and greater layer height resulted in increased surface unevenness due to imperfect polymer leakage during the 3D printing, which enhanced light transmission during diffuse light propagation through the transparent 3D-printed material samples. It was also observed (see Table 6) that increasing the printing temperature of samples produced with a 0.4 mm nozzle diameter and a 0.2 mm layer height improved their light transmission properties. This phenomenon is probably caused by internal defects and better layer bonding, reflected by reducing the number of microscopic gaps and irregularities that could otherwise scatter light. Consequently, increasing the 3D printing temperature creates a more homogeneous material structure, which enhances its light transmission properties as light propagates through this structure. The light transmittance values obtained for the investigated 3D-printed polymer samples were consistent with their microscopic images, as illustrated in Figure 3 and Figure 4.

#### 3.5.2. Haze

The haze values, which express the percentage of transmitted diffuse light scattered by more than 2.5°, are shown in Table 6. The measured haze values were primarily affected by the nozzle diameter rather than the layer height or printing temperature. Generally, the 3D-printed polymer samples manufactured with a nozzle diameter of 0.4 mm exhibited higher haze values than those produced with a nozzle diameter of 0.8 mm. As a result, the polymer specimens produced with a 0.4 mm nozzle diameter scattered diffuse light more significantly, reducing direct transmission and creating a hazy or cloudy appearance of objects seen through these materials. In contrast, the 3D-printed polymer samples manufactured with a 0.8 mm nozzle diameter transmitted light with less scattering, making objects behind them appear clear and sharp. These findings corresponded to the microscopic images of the investigated 3D-printed polymer specimens, as shown in Figure 3 and Figure 4.

## 4. Conclusions

This study investigated the impact of 3D printing conditions, namely nozzle diameter, layer height, and printing temperature, on the mechanical and physical properties of three polymer materials, PLA, PMMA, and PETG, produced using the FDM printing technique. The mechanical properties examined included bending and vibration damping properties, while the physical properties studied were sound absorption and light transmission of the tested 3D-printed specimens. The results indicate that the 3D printing conditions applied substantially affected the polymer specimens’ surface morphology and material structure, influencing their mechanical and physical characteristics.

This study revealed that the bending stiffness, characterized by the bending modulus, was higher (by 44 to 64%) in the case of the 3D-printed PLA specimens compared to the PMMA and PETG samples, which corresponded to Young’s modulus values of the filaments used to produce the 3D-printed samples. Generally, bending stiffness increased with a decrease in nozzle diameter and layer height, attributed to improved melting and interlayer adhesion, which reduces polymer degradation. These findings were confirmed by a non-destructive vibration damping method when a higher mechanical stiffness of the studied material samples was accompanied by a shift of the first resonance frequency peak position to higher frequency values. However, the increased printing temperature of the tested PLA sample led to mechanical degradation of its structure, which decreased its dynamic mechanical stiffness and the first resonance frequency. The non-destructive nature of the vibration damping method offers a key advantage over traditional destructive methods, such as tensile, compression, and bending tests. Additionally, bending tests indicated that PETG specimens exhibited greater ductility, demonstrating a higher capacity to absorb mechanical overloads than other polymers. The 3D cross-layer deposition printing technique used for specimen production enhanced the bending stiffness of the tested polymer materials compared to conventionally 3D-printed samples fabricated with varying raster angle orientations.

The 3D printing conditions significantly impacted the sound absorption properties of the manufactured samples, primarily due to surface quality. Samples printed with a smaller nozzle diameter and layer height generally displayed smoother surface textures, reducing sound absorption. Similarly, increased printing temperatures in PMMA and PETG samples led to a smoother structure with lower sound absorption capacity. In contrast, a higher printing temperature caused mechanical degradation in PLA, enhancing its sound absorption and vibration damping properties. Overall, the sound absorption of the printed polymer samples was relatively low, with an average sound absorption coefficient between 0.09 and 0.21. As a result, these 3D-printed samples are better suited for sound reflection applications, such as sound-insulating partitions in theaters, concert halls, and recording studios.

The applied 3D printing conditions also influenced the light transmission properties of the investigated polymer specimens. In general, diffuse light transmission increased with nozzle diameter and layer height. Higher printing temperatures enhanced layer bonding, improving light transmission, ranging from 7% in PMMA to 25% in PLA specimens. These results were consistent with the haze measurements, which showed that samples printed with a nozzle diameter of 0.8 mm transmitted light with less scattering, making objects behind them appear clear and sharp. Conversely, specimens with a nozzle diameter of 0.4 mm scattered more diffuse light, reducing direct transmission and creating a hazy or cloudy appearance of objects. In conclusion, the light transmission and sound absorption properties of the 3D-printed polymer specimens were consistent with their microscopic images.

This study highlights the importance of optimizing 3D printing parameters to enhance the mechanical and physical properties of polymer materials. Future research should focus on exploring these parameters further to develop comprehensive guidelines for optimizing 3D printing processes for various applications. By carefully selecting and controlling 3D printing conditions, it is possible to tailor the properties of 3D-printed materials to meet specific application requirements, thereby expanding the potential uses of 3D printing technology in various industries.

## Figures and Tables

**Table 1:** Basic parameters of filaments used for production of 3D-printed samples.

Parameter	Filament Material		
	PLA	PMMA	PETG
*d* (mm)	1.75 *	1.75 *	1.75 *
*E* (GPa)	3.60 *	1.80 *	1.95 *
*ρ* (g·cm^−3^)	1.24 *	1.19 *	1.27 *
*T*_1_ (°C)	205 ÷ 225 *	225 ÷ 250 *	230 ÷ 255 *
*T*_2_ (°C)	40 ÷ 60 *	80 ÷ 115 *	60 ÷ 85 *
Manufacturer	REGSHARE Ltd., (Horní Počaply, Czechia)	REGSHARE Ltd., (Horní Počaply, Czechia)	Spectrum Group Ltd., ((Pęcice, Poland)

* According to manufacturer’s data sheets.

**Table 2:** Designation and parameters of 3D-printed samples.

Sample Designation	Parameter				
	*D* (mm)	*H* (mm)	*T*_1_ (°C)	*T*_2_ (°C)	*ρ_s_* (g.cm^−3^)
PLA_0.8/0.4/215	0.8	0.4	215	60	1.18
PLA_0.8/0.2/215	0.8	0.2	215	60	1.19
PLA_0.4/0.2/215	0.4	0.2	215	60	1.21
PLA_0.4/0.2/235	0.4	0.2	235	60	1.14
PMMA_0.8/0.4/240	0.8	0.4	240	85	1.12
PMMA_0.8/0.2/240	0.8	0.2	240	85	1.13
PMMA_0.4/0.2/240	0.4	0.2	240	85	1.10
PMMA_0.4/0.2/260	0.4	0.2	260	85	1.12
PETG_0.8/0.4/240	0.8	0.4	240	85	1.24
PETG_0.8/0.2/240	0.8	0.2	240	85	1.23
PETG_0.4/0.2/240	0.4	0.2	240	85	1.18
PETG_0.4/0.2/260	0.4	0.2	260	85	1.22

**Table 3:** Mean values and corresponding standard errors of different quantities obtained from bending tests.

Sample Type	Parameter					
	*E_B_* (MPa)	*σ_max_* (MPa)	*ε_Fmax_* (%)	*ε_break_* (%)	*E*_σmax_ (mJ)	*E_break_* (mJ)
PLA_0.8/0.4/215	3219 ± 21	110.5 ± 0.3	4.3 ± 0.1	4.4 ± 0.2	800 ± 23	851 ± 74
PLA_0.8/0.2/215	3261 ± 140	112.2 ± 1.5	4.6 ± 0.1	4.9 ± 0.1	902 ± 15	1030 ± 32
PLA_0.4/0.2/215	3730 ± 66	107.3 ± 2.5	4.3 ± 0.1	5.1 ± 0.4	835 ± 29	1093 ± 133
PLA_0.4/0.2/235	3774 ± 31	112.5 ± 0.7	4.6 ± 0.1	5.7 ± 0.2	963 ± 5	1300 ± 77
PMMA_0.8/0.4/240	2070 ± 45	78.9 ± 1.0	5.3 ± 0.3	5.3 ± 0.3	704 ± 63	704 ± 63
PMMA_0.8/0.2/240	2258 ± 64	85.5 ± 0.7	6.3 ± 0.1	9.2 ± 0.7	1005 ± 16	1693 ± 165
PMMA_0.4/0.2/240	2277 ± 47	80.8 ± 0.1	5.1 ± 0.1	5.1 ± 0.1	712 ± 4	712 ± 4
PMMA_0.4/0.2/260	2345 ± 60	82.2 ± 0.4	5.3 ± 0.1	5.3 ± 0.1	759 ± 29	759 ± 29
PETG_0.8/0.4/240	2091 ± 32	86.5 ± 0.3	5.2 ± 0.1	−	774 ± 5	−
PETG_0.8/0.2/240	2173 ± 56	87.7 ± 0.1	5.2 ± 0.1	−	756 ± 22	−
PETG_0.4/0.2/240	2441 ± 29	85.7 ± 1.3	4.9 ± 0.1	−	727 ± 38	−
PETG_0.4/0.2/260	2385 ± 65	88.5 ± 0.6	4.9 ± 0.1	−	769 ± 10	−

**Table 4:** Mean values and corresponding standard deviations of the first resonance frequencies *f_R_*_1_ depending on printing conditions for investigated 3D-printed polymer samples loaded with an inertial mass *m_i_* = 90 g.

Sample Type	*f_R_*_1_ (Hz)
PLA_0.8/0.4/215	574 ± 19
PLA_0.8/0.2/215	1165 ± 34
PLA_0.4/0.2/215	1470 ± 29
PLA_0.4/0.2/235	749 ± 21
PMMA_0.8/0.4/240	480 ± 15
PMMA_0.8/0.2/240	897 ± 33
PMMA_0.4/0.2/240	1112 ± 28
PMMA_0.4/0.2/260	1125 ± 35
PETG_0.8/0.4/240	487 ± 13
PETG_0.8/0.2/240	841 ± 21
PETG_0.4/0.2/240	1351 ± 27
PETG_0.4/0.2/260	1299 ± 28

**Table 5:** Sound absorption properties of the studied 3D-printed specimens.

Sample Type	*α_m_* (−)	*NRC* (−)	*α_max_* (−)	*f_max_* (Hz)
PLA_0.8/0.4/215	0.209	0.114	0.610	2392
PLA_0.8/0.2/215	0.165	0.101	0.451	2304
PLA_0.4/0.2/215	0.132	0.072	0.267	2376
PLA_0.4/0.2/235	0.150	0.092	0.400	2240
PMMA_0.8/0.4/240	0.141	0.071	0.281	2608
PMMA_0.8/0.2/240	0.129	0.074	0.242	2720
PMMA_0.4/0.2/240	0.111	0.051	0.223	2984
PMMA_0.4/0.2/260	0.063	0.052	0.213	1568
PETG_0.8/0.4/240	0.166	0.065	0.426	2800
PETG_0.8/0.2/240	0.142	0.045	0.351	3448
PETG_0.4/0.2/240	0.122	0.049	0.255	2992
PETG_0.4/0.2/260	0.091	0.036	0.178	2984

**Table 6:** Mean values and corresponding standard deviations of the light transmittance *T* and haze *H* depending on printing conditions for investigated 3D-printed polymer samples.

Sample Type	*T* (−)	*H* (%)
PLA_0.8/0.4/215	0.66 ± 0.02	89.9 ± 0.1
PLA_0.8/0.2/215	0.67 ± 0.02	87.4 ± 0.1
PLA_0.4/0.2/215	0.52 ± 0.02	97.7 ± 0.1
PLA_0.4/0.2/235	0.64 ± 0.02	93.7 ± 0.1
PMMA_0.8/0.4/240	0.74 ± 0.03	84.6 ± 0.1
PMMA_0.8/0.2/240	0.78 ± 0.03	79.9 ± 0.1
PMMA_0.4/0.2/240	0.54 ± 0.02	95.3 ± 0.1
PMMA_0.4/0.2/260	0.58 ± 0.02	93.2 ± 0.1
PETG_0.8/0.4/240	0.65 ± 0.02	87.4 ± 0.1
PETG_0.8/0.2/240	0.59 ± 0.02	84.2 ± 0.1
PETG_0.4/0.2/240	0.37 ± 0.01	96.6 ± 0.1
PETG_0.4/0.2/260	0.44 ± 0.01	95.5 ± 0.1

## Data Availability

The data supporting this study’s findings are available in [Zenodo] at https://doi.org/10.5281/zenodo.14005312 (accessed on 24 November 2024) [77].
